# Supplementary material for: circFL-seq reveals full-length circular RNAs with rolling circular reverse transcription and nanopore sequencing
Source: eLife. 2021 Oct 14;10:e69457. doi: 10.7554/eLife.69457 (PMC8550772; doi:10.7554/eLife.69457)
Supplement: Figure 1—source data 1. — This file includes figures with uncropped gels. [file elife-69457-fig1-data1.zip › Figures_original_gels.pptx]

## Slide 1
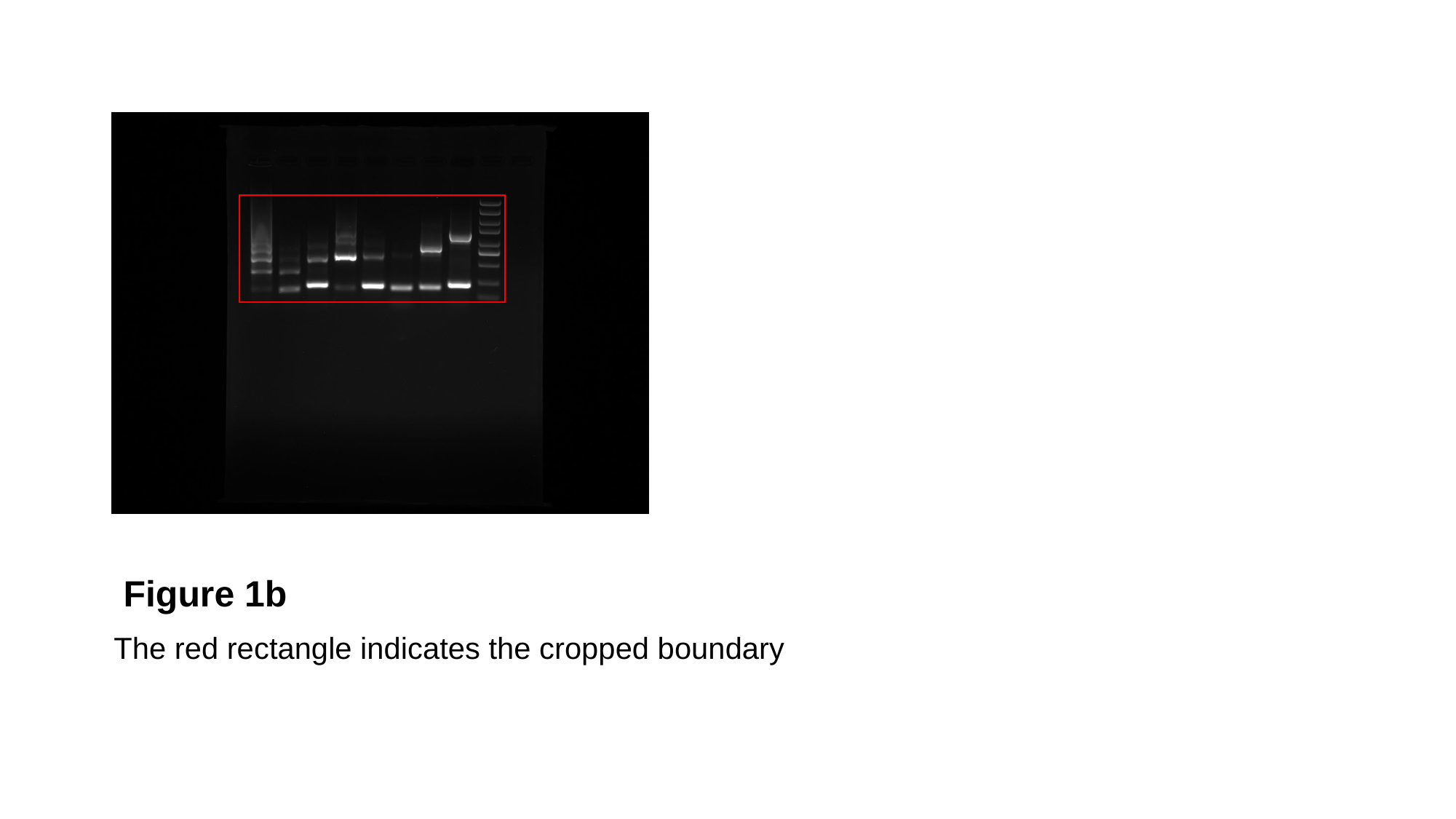

Figure 1b
The red rectangle indicates the cropped boundary

## Slide 2
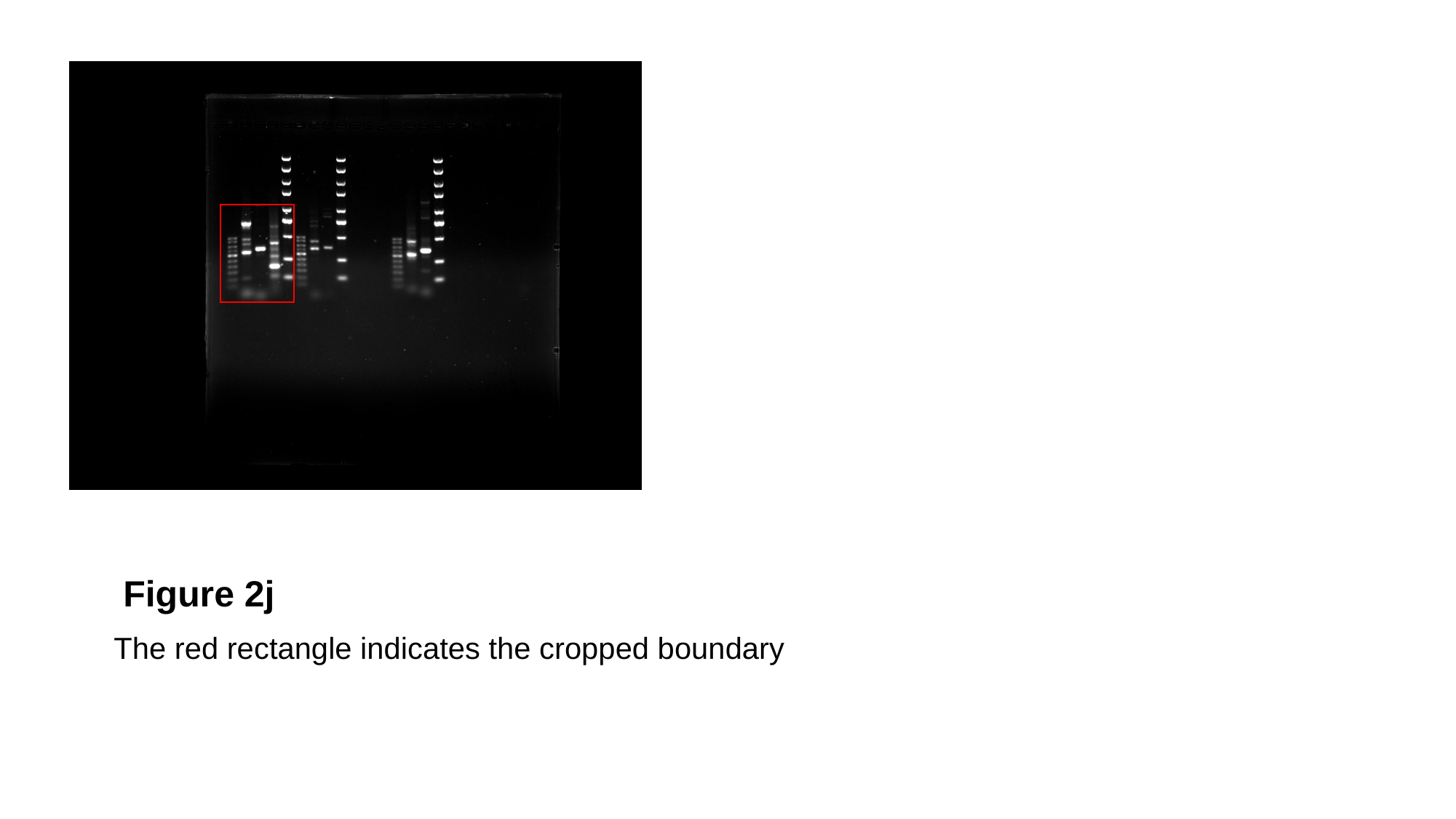

Figure 2j
The red rectangle indicates the cropped boundary

## Slide 3
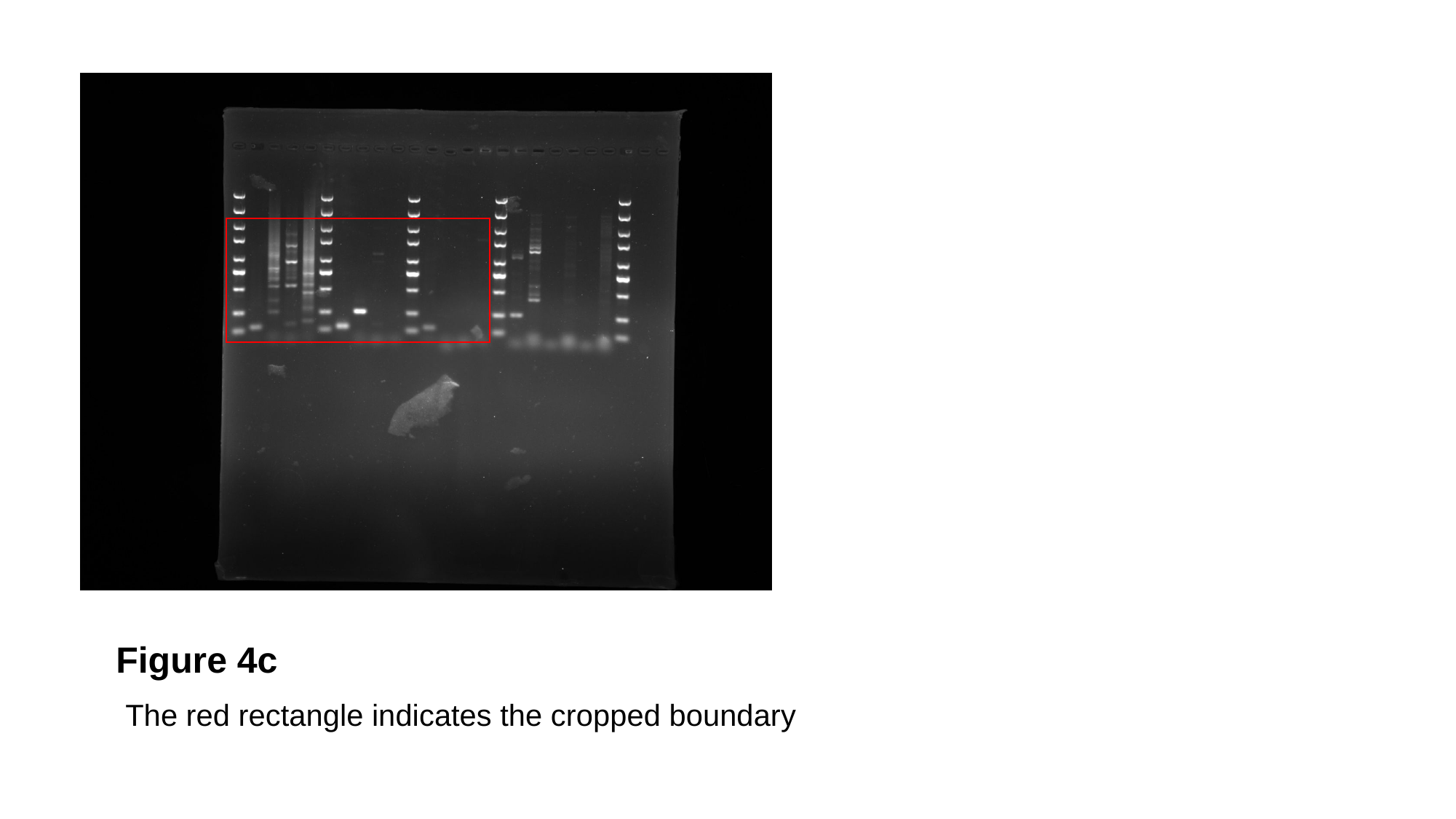

Figure 4c
The red rectangle indicates the cropped boundary

## Slide 4
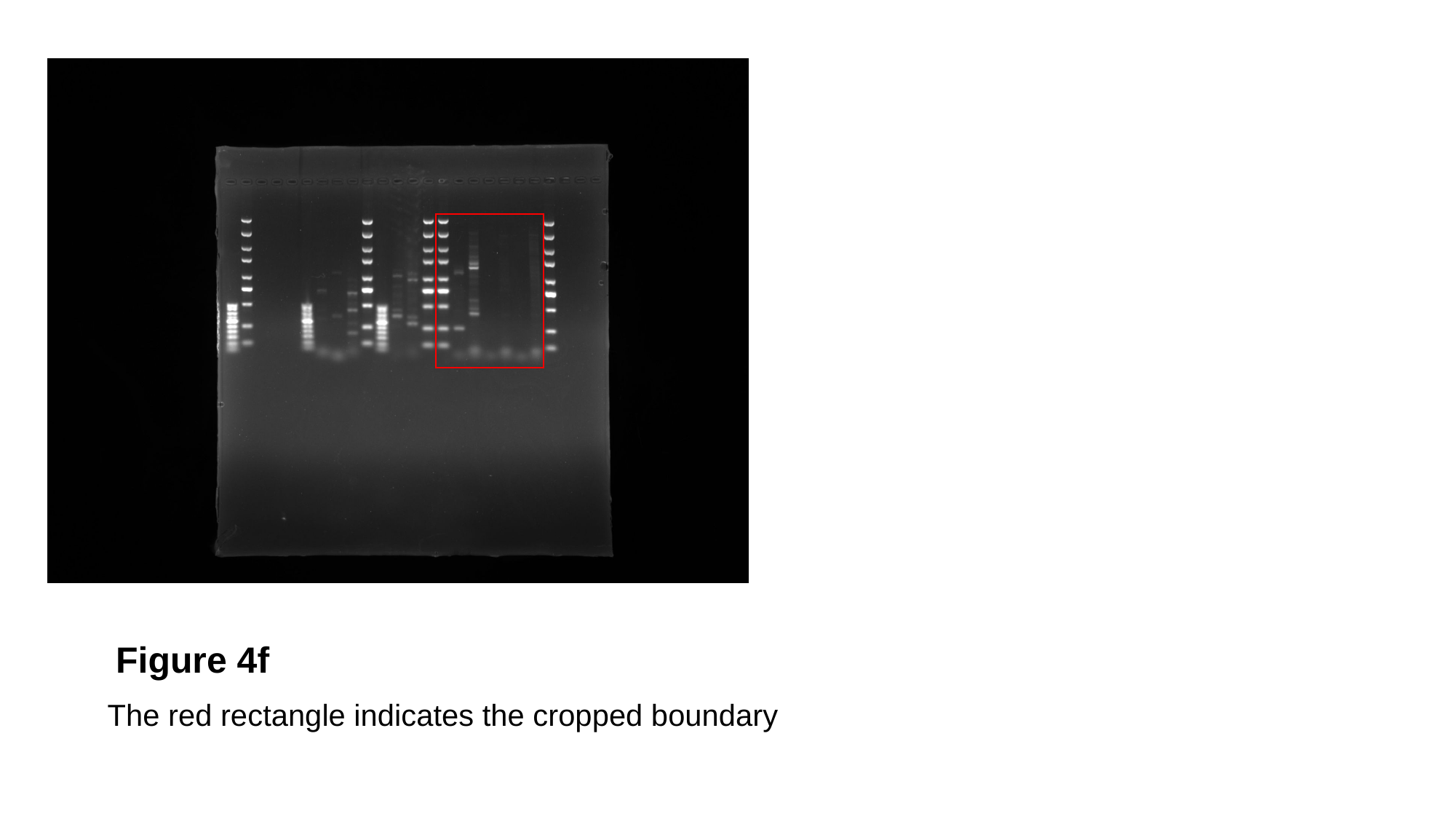

Figure 4f
The red rectangle indicates the cropped boundary
