# Supplementary figures and images for: circFL-seq reveals full-length circular RNAs with rolling circular reverse transcription and nanopore sequencing
Source: eLife. 2021 Oct 14;10:e69457. doi: 10.7554/eLife.69457 (PMC8550772; doi:10.7554/eLife.69457)

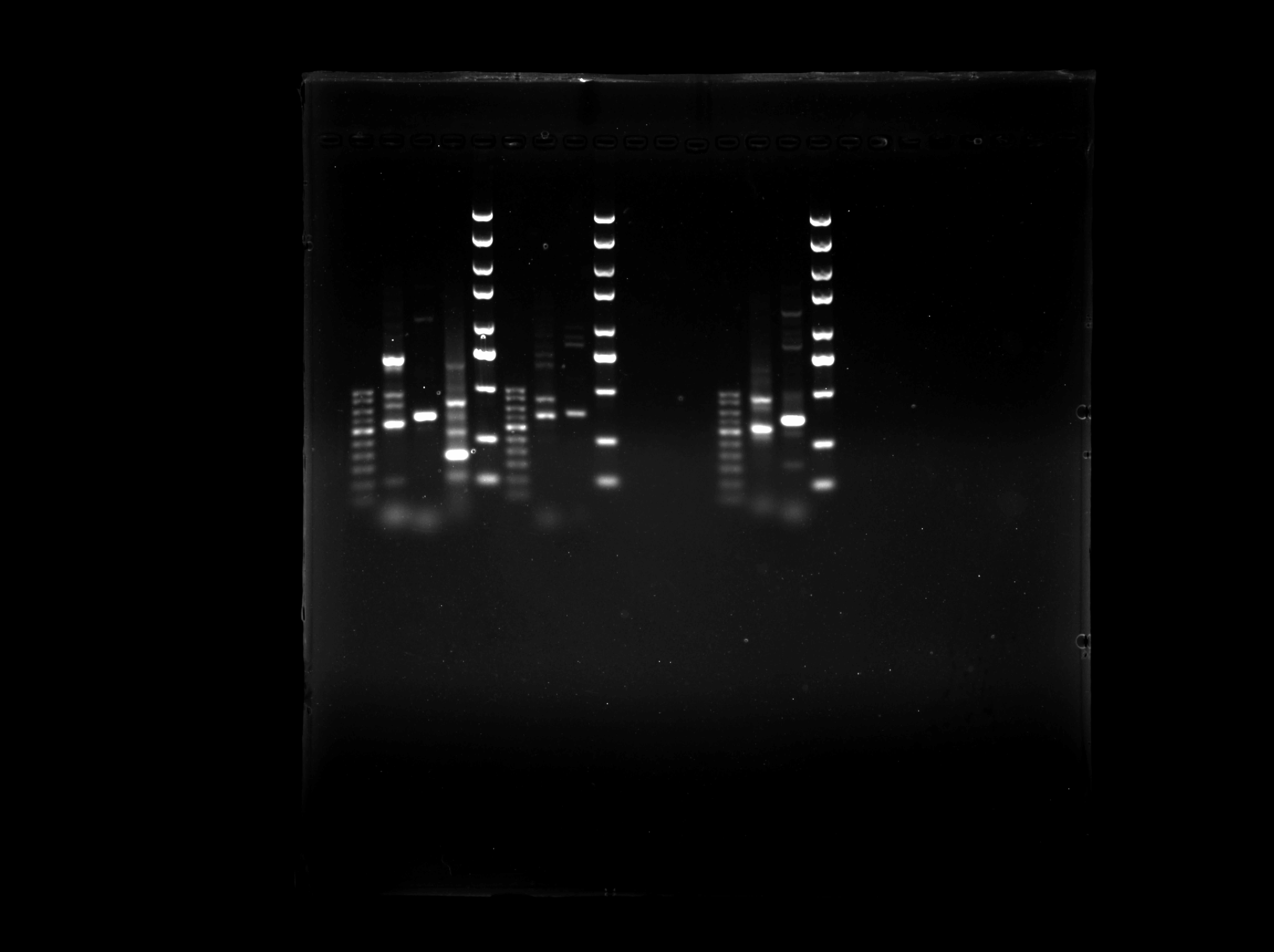

Supplement: Figure 1—source data 1. — This file includes figures with uncropped gels. [file elife-69457-fig1-data1.zip › Figure 2j.jpg]

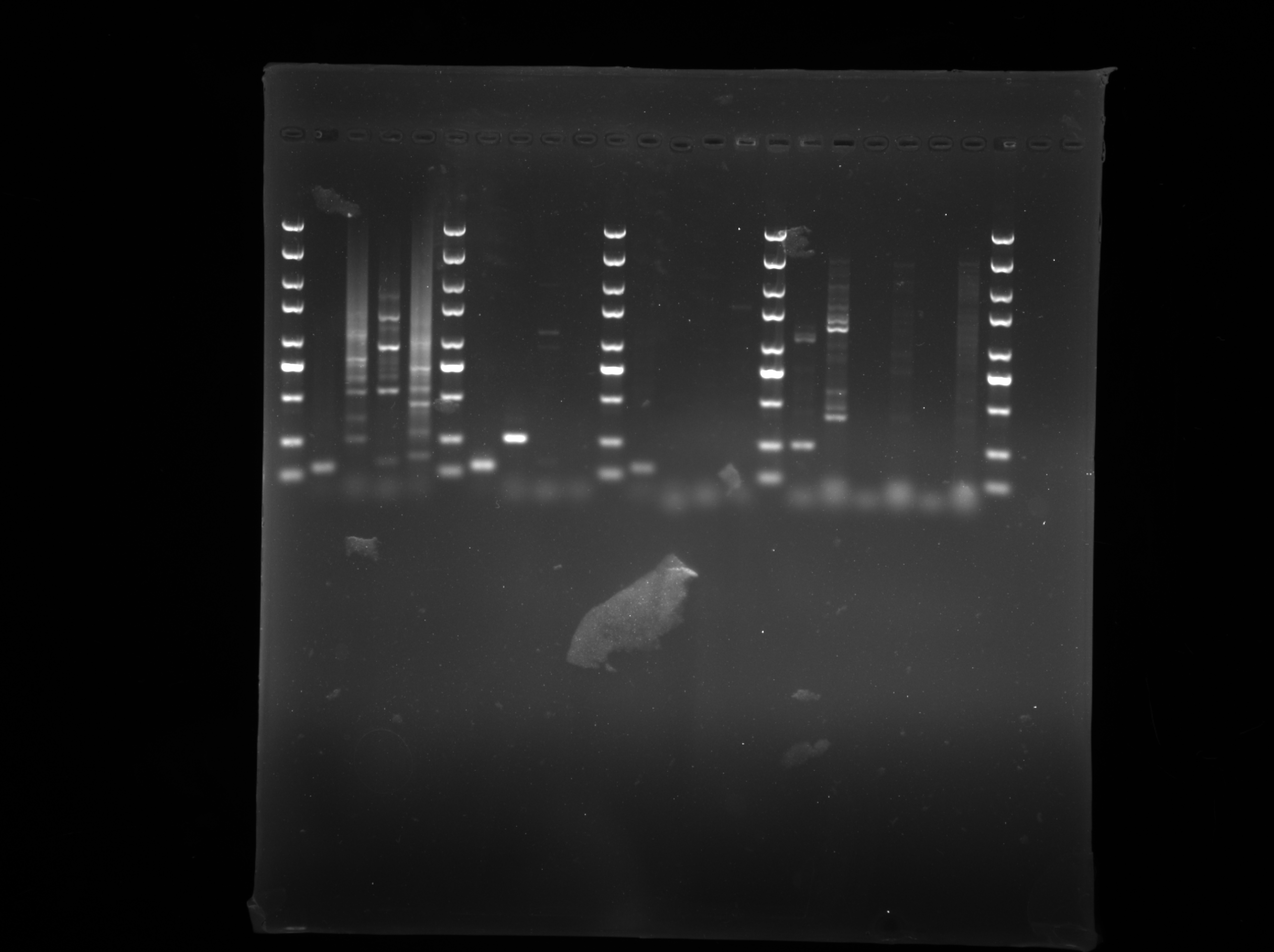

Supplement: Figure 1—source data 1. — This file includes figures with uncropped gels. [file elife-69457-fig1-data1.zip › Figure 4c.jpg]

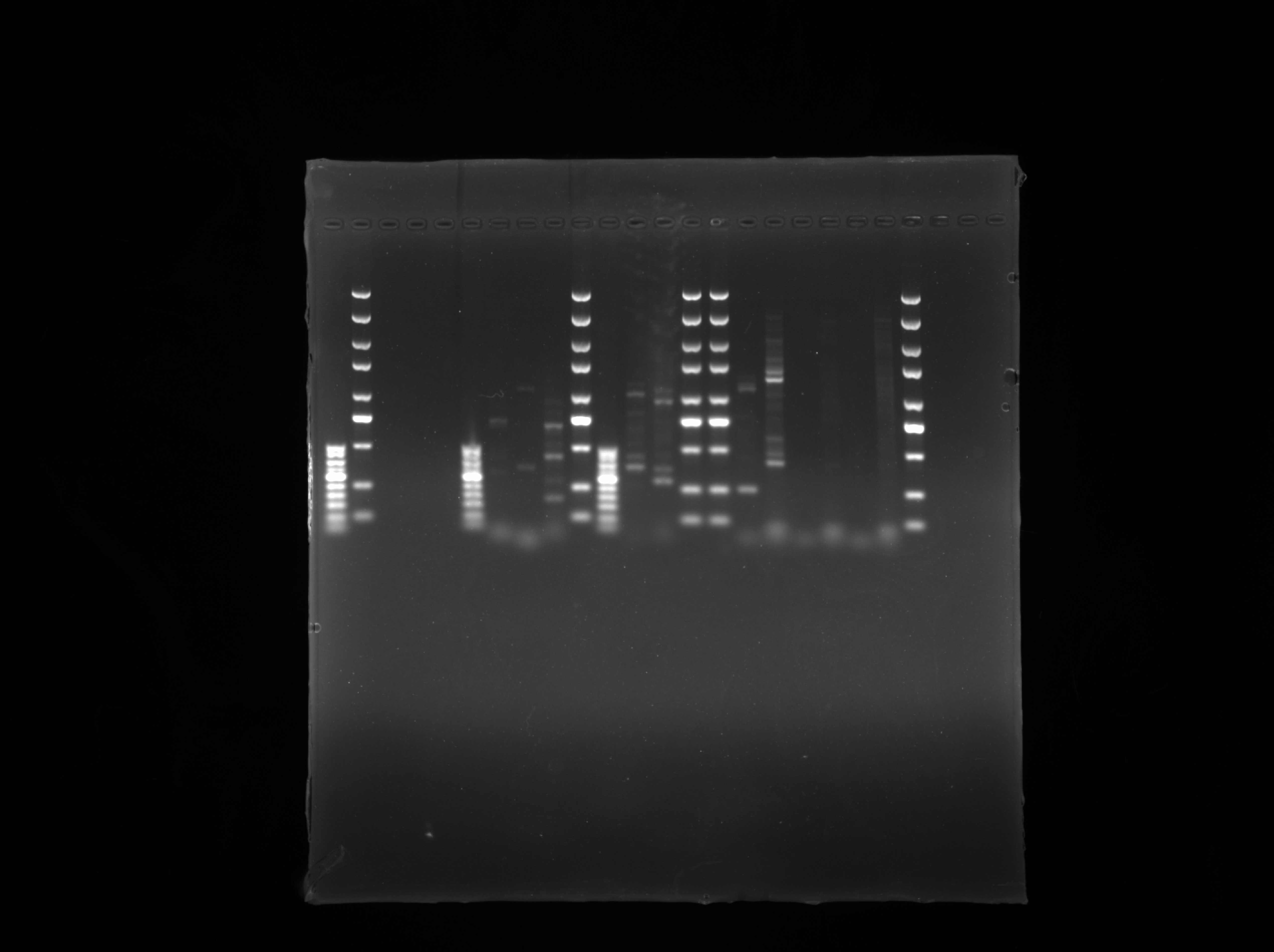

Supplement: Figure 1—source data 1. — This file includes figures with uncropped gels. [file elife-69457-fig1-data1.zip › Figure 4f.jpg]

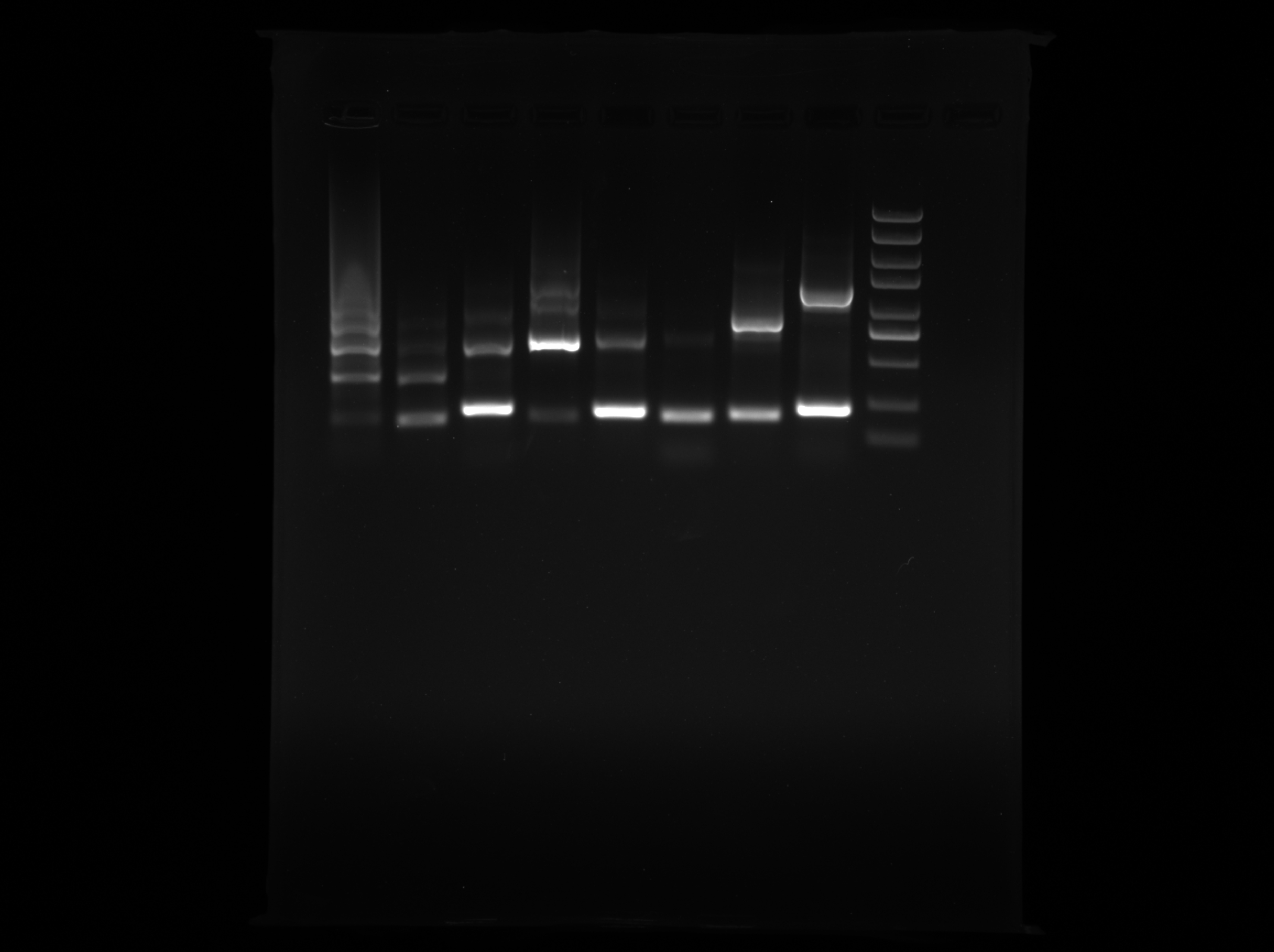

Supplement: Figure 1—source data 1. — This file includes figures with uncropped gels. [file elife-69457-fig1-data1.zip › Figure 1b.jpg]
