# Supplementary material for: circFL-seq reveals full-length circular RNAs with rolling circular reverse transcription and nanopore sequencing
Source: eLife. 2021 Oct 14;10:e69457. doi: 10.7554/eLife.69457 (PMC8550772; doi:10.7554/eLife.69457)
Supplement: Supplementary file 1. [file elife-69457-supp1.docx]

**Data summary of circFL-seq library**

| **barcode** | **sample ID** | **total reads (bases)** | **clean reads (bases)** | **full-length circRNA reads (bases)** | **# of circRNA BSJs** | **# of circRNA isoforms** | **# of BSJs annotated in database** |
| --- | --- | --- | --- | --- | --- | --- | --- |
| **PromethION of eight cell samples** | | | | | | | |
| barcode04 | HeLa rep1 | 37,426,448 (65,579,705,023) | 3,101,970 (2,209,357,508) | 68,788  (72,972,987) | 25,414 | 27,835 | 13,224 |
| barcode05 | HeLa rep2 |  | 4,327,013 (3,274,272,241) | 106,769  (119,557,697) | 38,566 | 43,193 | 19,493 |
| barcode02 | SKOV3 rep1 |  | 2,832,740 (2,334,866,989) | 159,919  (178,730,747) | 32,021 | 34,611 | 13,892 |
| barcode03 | SKOV3 rep2 |  | 4,112,952 (3,145,095,988) | 109,916  (115,056,684) | 34,079 | 36,603 | 15,410 |
| barcode08 | MCF7 |  | 4,674,823 (3,896,608,447) | 401,902  (451,686,680) | 28,764 | 35,541 | 20,425 |
| barcode07 | VCaP |  | 4,194,321 (3,521,440,797) | 224,036  (245,934,246) | 33,166 | 39,314 | 23,471 |
| barcode06 | SH-SY5Y |  | 3,919,978 (3,454,615,812) | 163,839  (194,665,036) | 26,844 | 31,242 | 18,552 |
| barcode01 | HEK293T |  | 2,853,078 (2,108,085,290) | 104,279  (111,672,860) | 22,165 | 25,305 | 16,710 |
| **MinION of HEK293** | | | | | | | |
| not applicable | HEK293 | 12,607,142 (10,430,206,187) | 7,493,857 (5,497,747,836) | 122,985  (135,309,549) | 27,869 | 32,985 | 20,991 |
| **PromethION of two human tissues** | | | | | | | |
| barcode04 | Human brain | 103,591,891  (102,142,860,701) | 41,617,172 (36,053,389,977) | 7,314,984  (7,547,402,867) | 33,042 | 49,865 | 18,875 |
| barcode03 | Human Testis |  | 25,793,620 (22,399,553,804) | 2,569,181  (2,714,805,610) | 43,700 | 56,748 | 25,769 |
