# Supplementary material for: circFL-seq reveals full-length circular RNAs with rolling circular reverse transcription and nanopore sequencing
Source: eLife. 2021 Oct 14;10:e69457. doi: 10.7554/eLife.69457 (PMC8550772; doi:10.7554/eLife.69457)
Supplement: Supplementary file 2. [file elife-69457-supp2.docx]

**Summary of alternative splicing events of circRNAs detected by circFL-seq**

| sample ID | circRNA with AS | | # of ES events | # of A3SS events | # of A5SS events | # of IR events |
| --- | --- | --- | --- | --- | --- | --- |
|  | # of BSJs | # of isoforms |  |  |  |  |
| HeLa | 3684 | 9190 | 2844 | 1498 | 1339 | 245 |
| SKOV3 | 3010 | 7184 | 2097 | 1270 | 1062 | 245 |
| MCF7 | 3714 | 8665 | 2408 | 1818 | 1587 | 179 |
| VCaP | 3707 | 8794 | 3021 | 1493 | 1279 | 194 |
| SH-SY5Y | 2726 | 6434 | 2069 | 1130 | 942 | 170 |
| HEK293T | 2077 | 4800 | 1577 | 733 | 676 | 100 |
| HEK293 | 3092 | 7378 | 2569 | 1124 | 915 | 170 |
| Human brain | 5350 | 15,187 | 3987 | 2810 | 2876 | 449 |
| Human Testis | 6673 | 15,546 | 5828 | 2708 | 3061 | 410 |

ES: Exon skipping; A3SS: Alternative 3’ splicing site; A5SS: Alternative 5’ splicing site; IR: Intron retention.
