# Supplementary material for: circFL-seq reveals full-length circular RNAs with rolling circular reverse transcription and nanopore sequencing
Source: eLife. 2021 Oct 14;10:e69457. doi: 10.7554/eLife.69457 (PMC8550772; doi:10.7554/eLife.69457)
Supplement: Supplementary file 3. [file elife-69457-supp3.docx]

**Data summary of RNA-seq library**

| **sample ID** | **# of clean reads** | **# of circRNA reads** | **# of circRNA BSJs** | **circRNA reads proportion** |
| --- | --- | --- | --- | --- |
| HeLa rep1 | 44,007,886 | 127,054 | 22,333 | 0.29% |
| HeLa rep2 | 43,058,726 | 134,436 | 23,800 | 0.31% |
| SKOV3 rep1 | 48,327,980 | 120,093 | 22,973 | 0.25% |
| SKOV3 rep2 | 51,460,908 | 166,262 | 26,987 | 0.32% |
| MCF7 | 49,544,998 | 155,219 | 22,029 | 0.31% |
| VCaP | 63,370,490 | 117,956 | 18,818 | 0.19% |
| SH-SY5Y | 56,364,304 | 158,874 | 22,146 | 0.28% |
| HEK293T | 69,105,154 | 103,384 | 17,028 | 0.15% |
