# Supplementary material for: circFL-seq reveals full-length circular RNAs with rolling circular reverse transcription and nanopore sequencing
Source: eLife. 2021 Oct 14;10:e69457. doi: 10.7554/eLife.69457 (PMC8550772; doi:10.7554/eLife.69457)
Supplement: Supplementary file 4. [file elife-69457-supp4.docx]

**Comparison of isoCirc and circFL-seq for circRNA detection in the HEK293 cell line**

| **sample** | **method** | **# BSJs** | **# known BSJs in database** | **# circRNA isoforms** | **# AS events for minor isoforms with read counts >1** | | | |
| --- | --- | --- | --- | --- | --- | --- | --- | --- |
|  |  |  |  |  | **ES** | **A3SS** | **A5SS** | **IR** |
| SRR10612050 | isoCirc | 39,410 | 16,260 | 41,516 | 194 | 66 | 54 | 41 |
| SRR10612051 |  | 42,372 | 17,115 | 44,767 | 217 | 86 | 53 | 51 |
| SRR10612052 |  | 55,058 | 20,797 | 58,303 | 343 | 134 | 91 | 77 |
| SRR10612053 |  | 50,621 | 24,048 | 54,876 | 428 | 155 | 110 | 72 |
| SRR10612054 |  | 52,164 | 24,318 | 56,567 | 488 | 150 | 108 | 83 |
| SRR10612055 |  | 38,547 | 19,293 | 41,483 | 302 | 87 | 87 | 53 |
| average |  | 46,362 | 20,300  43.8% | 49,585 | 329  55.8% | 113  19.2% | 84  14.3% | 63  10.7% |
| HEK293 | circFL-seq | 27,869 | 20,991  75. 3% | 32,985 | 994  64.4% | 273  17.7% | 199  12.9% | 77  5.0% |
