# Supplementary material for: circFL-seq reveals full-length circular RNAs with rolling circular reverse transcription and nanopore sequencing
Source: eLife. 2021 Oct 14;10:e69457. doi: 10.7554/eLife.69457 (PMC8550772; doi:10.7554/eLife.69457)
Supplement: Supplementary file 5. [file elife-69457-supp5.docx]

**Computational analysis of circFL-seq and CIRI-long**

| **sample** | **software** | **# BSJs** | **# known BSJs in database** | **# circRNA isoforms** | **# AS events for minor isoforms with read counts >1** | | | |
| --- | --- | --- | --- | --- | --- | --- | --- | --- |
|  |  |  |  |  | **ES** | **A3SS** | **A5SS** | **IR** |
| HEK293  circFL-seq | circFL-seq | 27869 | 20,991  75.3% | 32,985 | 994  64.4% | 273  17.7% | 199  12.9% | 77  5.0% |
|  | CIRI-long | 15242 | 11,725  76.9% | 16,118 | 220  71.0% | 22  7.1% | 19  6.1% | 49  15.8% |
| mouse  brain  CRR194208  CIRI-long | circFL-seq | 18396 | 12,615  68.6% | 20,309 | 461  75.9% | 63  10.3% | 51  8.4% | 32  5.3% |
|  | CIRI-long | 9258 | 6470  69.9% | 9525 | 50  66.7% | 5  6.7% | 6  8.0% | 14  18.7% |
