# Supplementary material for: circFL-seq reveals full-length circular RNAs with rolling circular reverse transcription and nanopore sequencing
Source: eLife. 2021 Oct 14;10:e69457. doi: 10.7554/eLife.69457 (PMC8550772; doi:10.7554/eLife.69457)
Supplement: Supplementary file 6. [file elife-69457-supp6.docx]

**Comparisons between circFL-seq, CIRI-long, and isoCirc.**

|  | **details** | **circFL-seq** | **CIRI-long** | **isoCirc** |
| --- | --- | --- | --- | --- |
| library construction | RCRT/RCA | RCRT | RCRT | RCA |
|  | size selection (kb) | ~1 | ~1 | 3-50 |
|  | second-strand synthesis | A tailing + anchor primer | templete switching | self-ligation to circular cDNA |
|  | amplification | PCR | PCR | RCA |
| tier-1 result | # circRNA full-length reads | **** | **** | ** |
|  | % circRNA full-length reads | ***** | ***** | ***** |
|  | read length | *** | *** | ***** |
|  | # rolling circles | *** | *** | ***** |
| tier-2 result | precision | **** | **** | *** |
|  | sensitivity | **** | *** | **** |
