# Supplementary material for: circFL-seq reveals full-length circular RNAs with rolling circular reverse transcription and nanopore sequencing
Source: eLife. 2021 Oct 14;10:e69457. doi: 10.7554/eLife.69457 (PMC8550772; doi:10.7554/eLife.69457)
Supplement: Supplementary file 8. [file elife-69457-supp8.docx]

**Summary of performance of strand classifier.**

| **sample ID** | **training size** | **test size** | **test accuracy** | **AUC** |
| --- | --- | --- | --- | --- |
| HeLa rep1 | 41,959 | 13,987 | 0.958 | 0.984 |
| HeLa rep2 | 66,626 | 22,209 | 0.96 | 0.987 |
| SKOV3 rep1 | 85,486 | 28,496 | 0.962 | 0.984 |
| SKOV3 rep2 | 62,364 | 20,788 | 0.964 | 0.987 |
| MCF7 | 247,764 | 82,588 | 0.969 | 0.994 |
| VCaP | 141,332 | 47,111 | 0.97 | 0.994 |
| SH-SY5Y | 98,078 | 32,693 | 0.965 | 0.989 |
| HEK293T | 67,431 | 22,477 | 0.967 | 0.991 |
| HEK293 | 69,427 | 23,143 | 0.982 | 0.994 |
| Human Testis | 1,769,854 | 589,952 | 0.978 | 0.996 |
| Human Brain | 4,781,710 | 1,593,904 | 0.984 | 0.997 |
